# Short-Term Biliary Stent Placement Contributing Common Bile Duct Stone Disappearance with Preservation of Duodenal Papilla Function

**DOI:** 10.1155/2016/6153893

**Published:** 2016-05-10

**Authors:** Tatsuki Ueda, Masataka Kikuyama, Yuzo Kodama, Takafumi Kurokami

**Affiliations:** ^1^Department of Gastroenterology, Kyoto University Hospital, 54 Kawaracho, Shogoin, Sakyoku, Kyoto 606-8507, Japan; ^2^Department of Gastroenterology, Shizuoka General Hospital, 4-27-1 Kita-ando, Aoiku, Shizuoka 420-8527, Japan

## Abstract

*Aims*. To investigate the effect of biliary stent placement without endoscopic sphincterotomy (EST) on common bile duct stones (CBDS) disappearance and the contribution of preserving the duodenal papilla function to reduce recurrence of CBDS.* Methods*. Sixty-six patients admitted for acute obstructive cholangitis due to CBDS who underwent biliary stent placement without EST for 2 years from March 2011 were evaluated retrospectively. The second endoscopic retrograde cholangiopancreatography (ERCP) was performed for treatment of CBDS 3 to 4 months after the first ERCP. We estimated the rate of stone disappearance at the time of second ERCP.* Results*. CBDS disappearance was observed in 32 (48.5%) of 66 patients. The diameter of the bile ducts and the diameter of CBDS in patients with CBDS disappearance were significantly smaller than in those with CBDS requiring extraction (*p* = 0.007 and *p* < 0.001, resp.). Stone disappearance was evident when the diameter of bile ducts and that of CBDS were <10 and 7 mm, respectively (*p* = 0.002).* Conclusions*. Short-term stent placement without EST eliminates CBDS while preserving duodenal papilla function and may be suitable for treating CBDS in patients with nondilated bile ducts and small CBDS.

## 1. Introduction

To treat common bile duct stones (CBDS), endoscopic sphincterotomy (EST) is an established procedure and is widely performed. However, late complications including liver abscess, cholangitis, CBDS recurrence, and bile duct cancer have recently been reported with this technique [1, 2], the occurrence of which is probably due to reflux into the bile duct of duodenal juice, which contains both pancreatic juice and bacteria. It is desirable to avoid such complications in younger patients, who have a long life expectancy.

Endoscopic papillary balloon dilation is an alternative method for treatment of CBDS [3], and it has the advantage of preserving duodenal papillary function [4]. However, compared with EST, a higher rate of postendoscopic retrograde cholangiopancreatography (ERCP) pancreatitis (PEP) has been reported [4], and this remains a potential hazard when using this method for treatment of CBDS.

Biliary stent placement is widely performed for acute obstructive cholangitis (AOC) due to CBDS. This procedure is easy to perform, effective, and accepted as an emergent treatment [5, 6]. In patients with difficult stones, biliary stent placement to drain obstructed bile juice due to CBDS can be selected [7–10]. Some reports have described a decrease in size and diameter, as well as disappearance of stones in patients with biliary stent placement after EST [7, 8].

We performed biliary stent placement in patients of various ages for AOC due to CBDS without EST during their first hospitalization. These patients were discharged temporarily after evidence of relief of AOC, and readmission for extraction of CBDS was scheduled 3 to 4 months after the first hospitalization. When endoscopic treatments were initiated at the second hospitalization, stone disappearance occurred in about half of patients. Herein, we report our findings in these patients.

## 2. Subjects and Methods

Sixty-six patients admitted for AOC due to CBDS who underwent biliary stent placement for 2 years from March 2011 were evaluated retrospectively. Patients with a past history of EST and biliary tract malignancies such as gallbladder carcinoma or bile duct carcinoma were excluded. Of the 66 patients, 43 were male and 23 were female. The mean age of these patients was 68.5 years (range: 36–94 years) (Table 1). Severity of AOC was confirmed in accordance with the 2013 Tokyo Guidelines [11]. Severity grades III, II, and I were noted in 3, 24, and 39 patients, respectively. Of the 59 patients with a gallbladder, 45 had gallbladder stones. This retrospective study was approved by the institutional review board of Shizuoka General Hospital.

Diagnosis of CBDS was confirmed by recognition of a movable filling defect on endoscopic retrograde cholangiopancreatography (ERCP), and a 7 Fr/7 cm double-pigtail stent (Olympus, Japan) was placed in all of these patients. Biliary stent placement was performed with a lateral-viewing endoscope (JF 260, Olympus, Japan). Bile duct diameter and CBDS were measured using ERCP images. After cannulating a bile duct, a small amount of contrast medium (60% Urographin, Bayer) was injected and CBDS was identified, followed by selective cannulation of the relevant bile duct. Bile juice was aspirated as much as possible, and a cholangiogram showing intrahepatic bile ducts and a cystic duct was recorded. After cholangiography, a 0.035-inch guide wire (Jagwire, Boston Scientific Japan) was inserted. The 7 Fr/7 cm double-pigtail biliary stent was placed over the guide-wire with the objective of fixing the tip of the stent to either hepatic duct. A pancreatic stent was placed simultaneously in cases where difficulty placing the cannula selectively extended the procedure time beyond 10 minutes, misinjection into the pancreatic duct occurred more than 3 times, or a small orifice in the major papilla was present.

Oral food intake was started on the day after stent placement, if symptoms of AOC such as pain, fever, and abnormal laboratory data were relieved. Patients were discharged temporarily if aggravation of AOC was not recognized after starting oral food intake. Readmission was scheduled for endoscopic treatment of CBDS 3 to 4 months after the first hospitalization. In patients with gallstones, cholecystectomy was performed before the second admission.

With the second ERCP, identification of CBDS was achieved by cholangiography while maintaining a biliary stent in the bile duct. When a filling defect revealing CBDS was absent, the biliary stent was removed and treatment for CBDS was terminated. On the other hand, when a filling defect showing CBDS was recognized, extraction of CBDS using a basket catheter was performed after removing the biliary stent with EST, or without EST because of small diameter of the stone. In patients treated with an anticoagulant, the biliary stent was maintained in place.

We estimated the rate of stone disappearance and compared diameters of bile ducts, diameters of CBDS, number of CBDS, ratio of calcified CBDS to total CBDS, and duration from discharge to second admission in the 2 groups (i.e., those with stone disappearance and stone persistence). Stone disappearance was confirmed by ERCP. Complications associated with endoscopic procedures were evaluated. The recurrence rate of CBDS after the second ERCP with an average follow-up period of 34.3 months (9–44 months) was estimated, while recurrence of CBDS was evaluated with recurrence of symptoms of cholangitis.

The data obtained in this study were statistically analyzed by Student's *t*-test and Fisher's exact test to determine factors related to stone disappearance. *p* values <0.05 were regarded as statistically significant.

## 3. Results

CBDS disappeared in 32 (48.5%) of 66 patients (Table 2). Diameters of the bile ducts and the diameter of CBDS in patients with stone disappearance (Table 3) were significantly smaller than in those without stone disappearance (*p* = 0.007 and *p* < 0.001, resp.). The number of stones, ratio of calcified stones, and duration from first hospitalization discharge to second admission were not significantly different between the 2 groups (*p* = 0.998, *p* = 0.180, and *p* = 0.205, resp.). Seventeen patients had bile duct and CBDS diameters of <10 and 7 mm, respectively (Table 4). CBDS disappeared in 14 (82.4%) of the 17 patients. When the diameters of the bile duct and stones were <10 and 7 mm, respectively, CBDS disappeared readily (*p* = 0.002).

With respect to complications (Table 5), mild post-ERCP pancreatitis and middle hepatic vein thrombosis were experienced in 4 (6.1%) patients and 1 (1.5%) patient, respectively. The latter complication was caused by compression of the middle hepatic vein by the tip of the stent placed at the caudal lobe.

**Figure 1:**
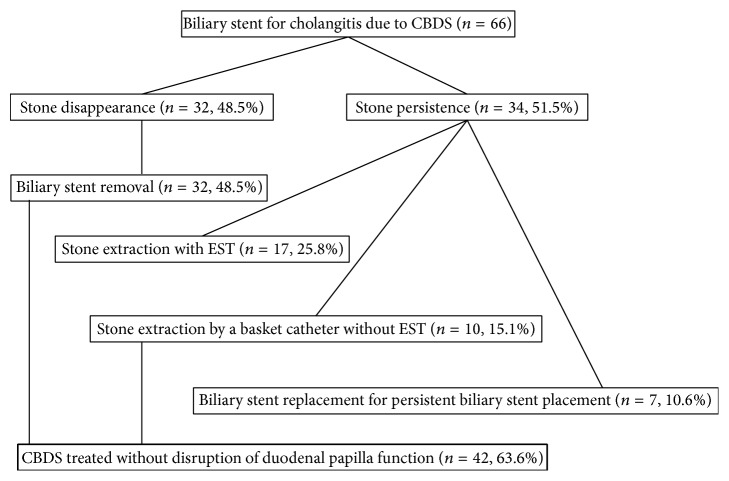
Flow diagram of patients analyzed in this study.

During the second ERCP (Table 6), 32 patients with stone disappearance underwent biliary stent removal and the treatment for CBDS was completed. Among patients without stone disappearance, stone extraction using a basket catheter with EST was performed in 17 (25.8%) patients and stone extraction using a basket catheter without EST because of small stone diameter and a widely opened papilla orifice due to stent placement (phenomenon which was frequently experienced in our study) was performed in 10 (15.1%) patients. Biliary stent replacement, for persistent biliary stent placement due to a large stone, and anticoagulant administration were performed in 7 (10.6%) patients. Finally, CBDS were treated without disruption of duodenal papilla function in 42 (63.6%) patients (Figure 1).

During an average follow-up period of 34.3 months (Table 6), cholangitis due to stone recurrence was experienced in 1 (3.1%) patient with stone disappearance, 2 (11.8%) patients with EST, and 1 (10%) patient with stone extraction without EST. However, the recurrence rate of cholangitis was not significantly different between the two groups: stone disappearance or stone extraction by a basket catheter without EST and stone extraction with EST (*p* = 0.57).

## 4. Discussion

Several studies have investigated stent placement for the treatment of CBDSin cases with stones that cannot be removed by ordinary endoscopic treatments including EST [7–10]. In one report, plastic stent placement was evaluated for the treatment of large CBDS in 45 patients [7]. Among all 45 patients, EST was performed in cases where extraction of CBDS failed. A decrease in the size of stones was observed in almost all patients and CBDS disappearance occurred in 22.2% of patients. Agitation of CBDS within the bile duct where the stent was located was suggested to be the most likely explanation for these results. In another report [8], placement of a pigtail stent after EST resulted in disappearance in 7 (35%) of 20 patients and a decrease in the size of CBDS in 11 (55%) of 20 patients, presumably caused by grinding of the placed stent against the stone.

Stent placement for 2 months was reported to contribute to a decrease in the number and size of CBDS in almost all of a cohort of 40 patients who did not undergo EST [9]. CBDS diameters were greater than 20 mm in many patients; moreover, disappearance of small CBDS was observed. The authors presumed that disruption of stones caused by the placed stent contributed to a decrease in CBDS diameters and numbers. Friability of stones was also described as a possible mechanism over the short term, resulting in facilitation of endoscopic procedures using a lithotriptor for CBDS [12]. Agitation, grinding, and friability of stones might contribute to the destruction of CBDS, enabling them to be discharged, and preserving duodenal papilla function in patients who have not undergone EST is also considered to be responsible for disappearance of small CBDS by facilitating their discharge.In another report, discharge of CBDS with preservation of the duodenal papilla was observed during ERCP [13]. This suggests that discharge of CBDS is a possible mechanism for CBDS disappearance.

In our study, CBDS disappeared with short-term stent placement for about 3 months without EST in about 50% of patients. Smaller sizes of CBD and CBDS were associated with CBDS disappearance. Short-term stent placement could contribute to CBDS disappearance, especially in patients with CBD diameters of <10 mm and CBDS diameters of <7 mm. The most likely mechanism of the disappearance in our cases was discharge of CBDS through the functional duodenal papilla, in addition to agitation caused by the placed stent. This is because the smaller CBDS would be discharged more readily through the duodenal papilla and the smaller bile duct could be associated with preserved bile excretion function of the duodenal papilla and probably with CBDS discharge.

Previous studies differ from ours in that the endoscopic treatments used for patients in those studies, including EST, were not successful in removing the stones. In our patients, we did not attempt to remove CBDS by EST before stent placement, and we did not always encounter stones that were difficult to treat. These factors could have contributed to the higher rate of stone disappearance in our study compared with previous reports.

Although EST is widely performed to treat CBDS, complications including perforation, hemorrhage, and pancreatitis are experienced during or after the procedure[14, 15]. Moreover, disruption of the duodenal papilla lets the duodenal juice, including pancreatic juice and bacteria, reflux into the bile duct, which can induce liver abscess, cholangitis, and CBDS recurrence as late complications [1, 2]. Our results showed that recurrent cholangitis during an average follow-up period of 34.3 months occurred in 11.8% of patients with EST as the second ERCP procedure, while, compared with a group of patients who did not undergo EST because of stone disappearance or stone extraction without EST following biliary stent placement, the recurrence rate of cholangitis was not significantly different. However, the follow-up period was too short to estimate complications correctly.

Our method compelled patients to be admitted twice. In spite of the fact, the advantage of our method is the preservation of duodenal papilla function. Moreover, if the size of bile duct and CBDS is <10 and 7 mm, respectively, the disappearance rate is high and statistically significant. Patients that meet these criteria have the potential to experience great benefit with the procedure. In particular, younger patients with bile duct size and CBDS <10 and 7 mm, respectively, may be ideal candidates, because they have a long life expectancy and avoiding destruction of the duodenal papilla function would therefore be highly desirable.

We propose the following new strategy for CBDS treatment. For younger patients with CBD diameters of <10 mm and CBDS diameters of <7 mm, a biliary stent is placed temporarily for 3 months and a second ERCP is performed. If the CBDS disappears, the treatment is considered complete and the stent is removed. Patients with relatively large persistent stones would be indicated to undergo EST with stone extraction. In those with small but persistent stones, EST would not be needed for stone extraction.

We deemed that stones had disappeared by confirming the absence of a filling defect on ERCP. This is a typical method used to judge stone disappearance. Persistent stones cannot be excluded completely by ERCP if they are small in diameter. However, our results showed that even if small stones remained that could not be confirmed on ERCP, they would not be of clinical importance, because no cases of cholangitis occurred after the second ERCP in patients whose stones were judged to have disappeared.

## 5. Conclusions

Our study suggests that short-term stent placement without EST is effective for the treatment of CBDS with preservation of duodenal papilla function. Suitable initial candidates for this method of treatment are patients with nondilated bile ducts and small CBDS. Further study is warranted to confirm our results, because this study was limited by a small sample size and was performed at a single center.

## Figures and Tables

**Table 1:** Patient characteristics (*n* = 66).

Characteristics	*n*
Male/female	43/23
Mean age (years)	68.5 (36–94)
Cholangitis	
Grade III/II/I	3/24/39
Gallbladder with/without gallstones	45/14

**Table 2:** Stone disappearance (*n* = 66).

Stone disappearance/persistence (*n*)	32/34
Stone disappearance rate (%)	48.5

**Table 3:** Analysis of factors associated with stone disappearance and persistence (*n* = 66).

	CBDS disappearance (*n* = 32)	CBDS persistence (*n* = 34)	*p* value
Diameter of bile ducts (mm)	9.59 ± 3.43	12.20 ± 4.17	0.007
Diameter of bile duct stones (mm)	5.77 ± 3.01	11.21 ± 6.42	<0.001
Number of bile duct stones (pieces)	1.56 ± 0.98	1.55 ± 1.02	0.988
Ratio of calcified stones (%)	75.8	91.1	0.180
Duration from first hospitalization discharge to second admission (days)	143 ± 10	111 ± 58	0.205

**Table 4:** Stone disappearance in patients with CBD < 10 mm and CBDS < 7 mm.

	Diameter		*p* value
	CBD < 10 mm and CBDS < 7 mm	CBD ≥ 10 mm or CBDS ≥ 7 mm	
CBDS disappearance	14/17 (82.4%)	18/49 (36.7%)	0.002

**Table 5:** Complications.

Complications	*n* (%)
Mild pancreatitis	4 (6.1)
Hepatic vein thrombosis	1 (1.5)

**Table 6:** Procedures during the second ERCP and cholangitis after the second ERCP.

	Procedures during the second ERCP	*n* (%)	Cholangitis
			*n* (%)
Stone disappearance	Biliary stent removal	32 (48.5)	1 (3.1)^#^

Stone persistence	Stone extraction with EST	17 (25.8)	2 (11.8)^#^
	Stone extraction by a basket catheter without EST	10 (15.1)	1 (10)^#^
	Biliary stent replacement for persistent biliary stent placement	7 (10.6)	3 (42.9)

^#^ *p* = 0.57.

# shows statistical significance between “Biliary stent removal” plus “Stone extraction by a basket catheter without EST”, and “Stone extraction with EST”.
